# Targeted Minimally Invasive Percutaneous Posteromedial Release of Residual Clubfoot in Myelomeningocele Patients: A Report of Two Cases

**DOI:** 10.7759/cureus.27084

**Published:** 2022-07-20

**Authors:** Abdulmonem Alsiddiky, Naief Alghnimei, Rheema Alfadhil, Sultan K Alharbi, Abdulrahman M Alsharidah, Nora Albusayes, Reema Albarrak, Abdulaziz A Alsubaie

**Affiliations:** 1 Department of Orthopaedics, King Saud University, Riyadh, SAU; 2 Orthopaedics, King Salman Military Hospital, Tabuk, SAU; 3 Department of Orthopaedics, Majmaah University, Riyadh, SAU; 4 College of Medicine, King Saud University, Riyadh, SAU

**Keywords:** myelomeningocele, percutaneous release, release, minimally-invasive, clubfoot, mmc

## Abstract

Congenital talipes equinovarus (CTEV) is commonly associated with myelomeningocele (MMC). It is thought to be a mixture of intrauterine development and a result of an imbalance in muscular innervation. Conservative management has been explored for those cases, but most resulted in recurrence. In this study, we report two cases where targeted minimally invasive percutaneous posteromedial release of residual clubfoot was done using an 18-gauge needle and a small incision for the cuboid osteotomy. In both cases, we achieved plantigrade shoeable/braceable feet. Both cases are still followed in our clinic. They require further follow-up to assess their long-term outcomes.

## Introduction

Clubfoot, scientifically defined as congenital talipes equinovarus (CTEV), is a deformity that has a debatable and challenging treatment, especially when associated with syndromes [1]. According to a recent epidemiological study, it affects 150,000-200,000 newborn babies every year worldwide, with most cases occurring in developing countries [2]. This carries a great burden on the quality of life in the affected population and the community. Clubfoot has four complex foot abnormalities that constitute the deformity, which are forefoot adductus, midfoot cavus, hindfoot varus, and ankle equinus [3]. Talipes equinovarus could be either a congenital deformity with an unknown etiology (idiopathic) or a non-idiopathic deformity associated with other conditions, such as myelomeningocele (MMC), arthrogryposis, cerebral palsy, and poliomyelitis [1]. Non-idiopathic clubfeet account for 1.1/1000 to 1.6/1000 live births [4], and clubfoot is estimated to affect 2.3/1000 live births in Saudi Arabia [5]. Foot and ankle deformities are commonly associated with MMC as 32% of the foot and ankle MMC-associated deformities are clubfeet [6], and 40% of MMC children have clubfeet, making CTEV the most common foot deformity in MMC [7].

MMC (spina bifida) is the most common neural tube defect. It is caused by a defect in the posterior aspect of the vertebral column along with the overlying skin, which in turn exposes the meninges and spinal cord to the environment [8,9]. Deformity and neurological deficits in MMC differ depending on the level of the lesion, and clubfoot can occur at different levels; however, a mid-lumbar deficit at the level of L3 and L4 is the most commonly described one [10].

CTEV in MMC is thought to start during the intrauterine life by early foot agenesis, while the final result of muscular contractions and fibrosis is attributed to the loss of normal intrauterine dynamical muscle movements [6]. It is also possible for patients of MMC to develop clubfoot deformity as sequelae of the long-standing neuromuscular deficits [11]. More importantly, this may occur as a result of muscular imbalance where there is a lack of antagonism between the functioning innervated muscles and denervated muscles, the spastic effect of denervated muscles, or the effect of long-standing weight-bearing [7,10,11]. Other congenital deformities often seen with MMC include kyphosis, hemivertebrae, teratologic hip dislocation, and vertical talus [8]. Management of MMC-associated clubfoot presents much more of a challenge when compared to the idiopathic CTEV as it is combined with both motor and sensory dysfunctions [12].

Available treatment approaches include conservative management with serial manipulation and casting as well as multiple surgical options. Conservative methods are still considered effective and have been used for more than four decades [13-15]. The two most commonly used conservative techniques are the Ponseti method (PM) and the French functional method (FFM), both of which produced similar outcomes [16,17]. Both these methods include manipulation, stretching, and serial casting to achieve correction and prevent recurrence [17]. Manipulation in the PM involves correction of the deformities involved, which involves midfoot cavus, forefoot adduction, hindfoot varus, and ankle equinus. Correction is done in a stepwise manner, addressing each of the aforementioned deformities. Generally, idiopathic clubfoot responds well to conservative management. On the contrary, a non-idiopathic deformity is notably rigid and severe; hence, it does not respond to conservative management as well as the former and requires surgery most of the time [4,11].

Posteromedial soft tissue release is the classically adopted approach in syndromic non-idiopathic clubfoot [10]. This approach achieves the correction in a similar pathway to the conservative methods, where the forefoot adductus, midfoot cavus, and hindfoot varus are handled first followed by the correction of the ankle equinus deformity [16].

Although the conservative methods are considered well-established and successful modes for correction of clubfeet, they have a high recurrence rate when used alone in the management of non-idiopathic clubfeet [7,11]. Dunkley et al. reported a 40% recurrence rate of non-idiopathic clubfoot patients managed with the PM requiring additional management and 26% of those mandated surgical correction [18]. Arkin et al. reported that 57.7% feet in MMC patients had recurrent deformities after being managed with the PM requiring surgical management [19].

## Case presentation

Case 1

A five-year-old Syrian boy, who is a known case of spina bifida at the level of L3-L4 (low lumbar level MMC, according to Sharrard’s classification), was referred to the Pediatric Orthopedic Clinic at King Saud University Medical City as a case of congenital bilateral foot deformities. Perinatal history revealed that the mother was following regularly with obstetrics where spina bifida was discovered. The mother denied the use of supplements before or during her pregnancy and denied suffering from any chronic medical conditions. There was no similar health condition in the siblings or other members of the family. The child was born at the age of 40 weeks. He was admitted to the neonatal intensive care unit for observation and was awaiting a referral for the repair of spina bifida. The infant underwent several surgeries including the placement of a ventriculoperitoneal (VP) shunt at the age of 45 days and spina bifida repair at the age of three months. Both were uneventful, and he was discharged in a good condition. His bilateral foot deformity was not addressed during the same admission due to social reasons. 

**Figure 1:**
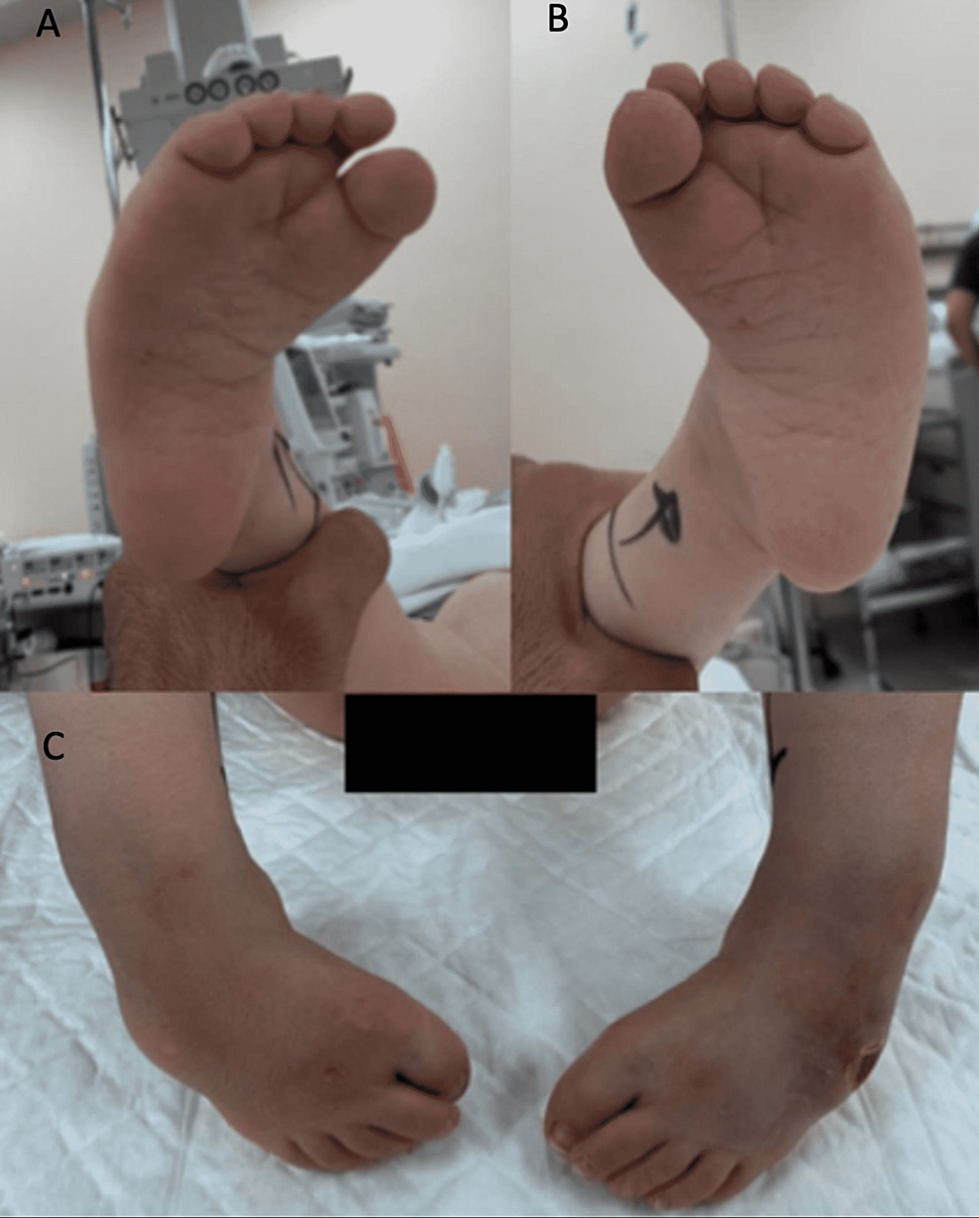
Preoperative appearance of the bilateral foot deformity in Case 1 (A) Preoperative appearance of the right foot deformity. (B) Preoperative appearance of the left foot deformity. (C) Preoperative appearance of the bilateral foot deformity.

His first visit to our clinic was when he was five years old. He was completely dependent on his motor function and had no formal physical therapy done nor was he enrolled in school. His general examination showed a communicative and joyful child with no dysmorphic features. His upper extremities were unremarkable. His spinal exam showed a moderate scoliosis deformity and a surgical scar over the previously repaired spina bifida. His hip exam revealed stable hip joints bilaterally with no leg length discrepancy noted, but his imaging revealed bilateral hip dislocations. His knee examination showed full active extension with no active flexion. His foot examination revealed bilateral rigid uncorrectable CTEV with a Pirani score of 5.5 with no active movements. There were 3-cm pressure ulcers on the dorsum of both feet. These ulcers were classified as stage 3 according to the National Pressure Injury Advisory Panel (NPIAP) and were treated with frequent dressing and local wound management. He had a loss of sensation below the level of L4. The child was able to pull to stand with his feet in an inverted position and therefore was bearing weight on the lateral aspect of the dorsum of his feet (see Figure 1).

**Figure 2:**
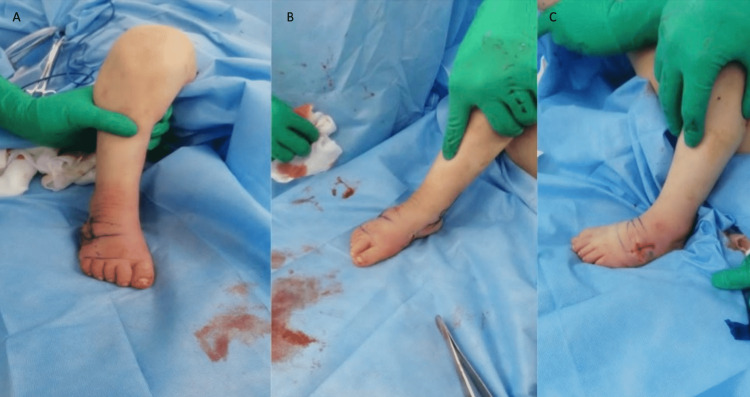
Intraoperative pictures of the foot after correction in Case 1 (A) Anterior view of the foot intraoperatively after correction. (B) Oblique view of the foot intraoperatively after correction. (C) Oblique view of the foot intraoperatively after correction.

Upon discussing the surgical option with the parents, the consensus that was reached was to reestablish painless plantigrade braceable feet. The consent was obtained for open versus percutaneous soft tissue releases and foot deformity reconstruction. The child was electively taken to the operating room where the patient was prepped and draped in the usual sterile manner, under general anesthesia, and in the supine position. Initially, examination under anesthesia was done to assess the deforming forces and feel for tight structure. We sequentially started to percutaneously release structures using an 18-gauge needle. With each structure released, we re-examined the foot for the need to release further. We started with the Achilles tendon, tibialis posterior (proximal to its insertion on the navicular), flexor hallucis longus, and, lastly, the plantar fascia (by deep palpation of the deforming force by abducting and dorsiflexing the foot). The tight posterior capsule was then addressed percutaneously from the posterolateral aspect of the Achilles tendon under fluoroscopic guidance. After finishing the posteromedial soft tissue release, examining the causes of inferomedial deforming forces, and eliminating the soft tissues, we proceeded to correct the metatarsus adductus, which was bony in origin. A corrective osteotomy over the lateral side of the cuboid bone was done, making a decancellation cuboid wedge osteotomy until reaching the lateral cuneiform. After this procedure, the examination revealed fully corrected feet with no skin tension. Wounds were irrigated with saline and closed. Above-knee casting was applied in the corrected position for four weeks postoperatively. Unfortunately, we were not able to obtain the preoperative and intraoperative x-ray images (see Figure 2).

**Figure 3:**
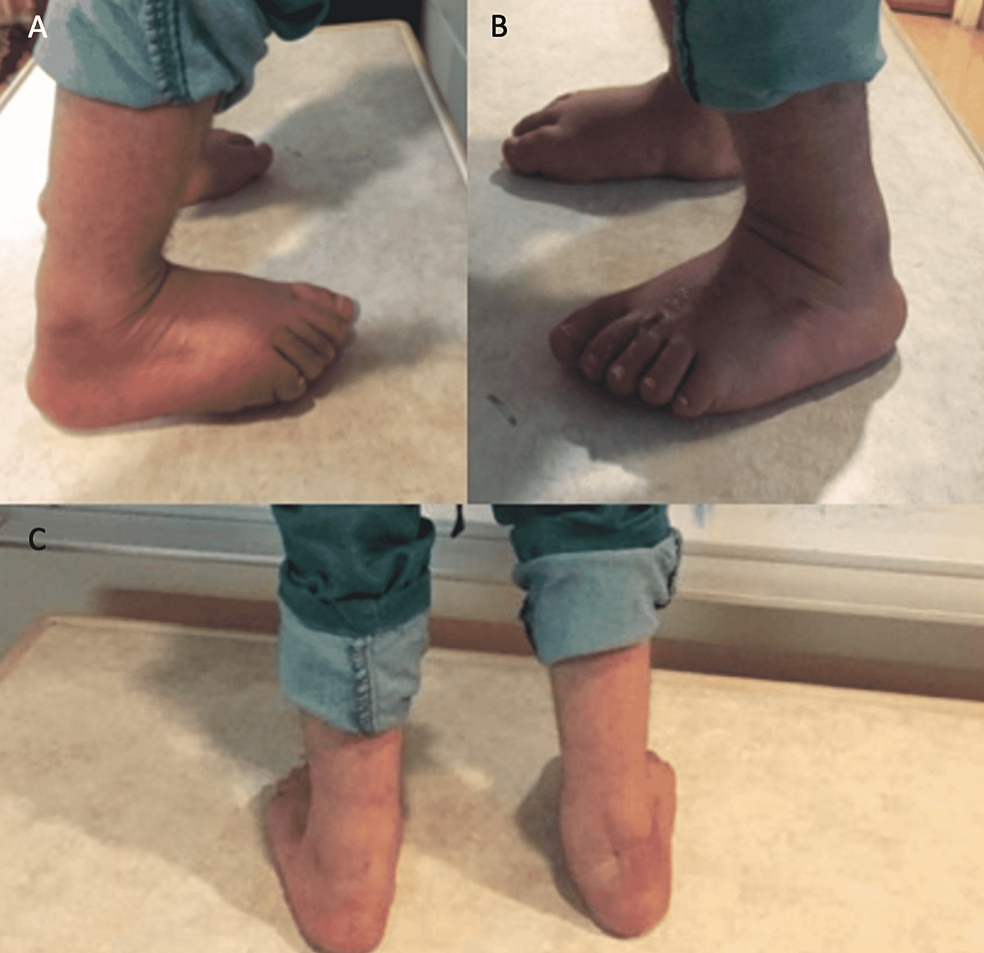
Postoperative appearance of the bilateral foot deformity at three months in Case 1 (A) Lateral view of the right foot deformity postoperatively after cast removal. (B) Lateral view of the left foot deformity postoperatively after cast removal. (C) Posterior view of the bilateral foot deformity postoperatively after cast removal.

Recently, the same patient underwent bilateral open reduction, bilateral varus derotation osteotomy, and hip spica casting for his bilateral hip dislocations. Currently, the patient is doing well. He is off the broomstick cast and is using the ankle foot orthosis (AFO) and continuing physical therapy. He is able to stand with assistance on plantigrade feet at three months postoperatively. Unfortunately, we were not able to obtain his postoperative x-ray images (see Figure 3).

Case 2

**Figure 4:**
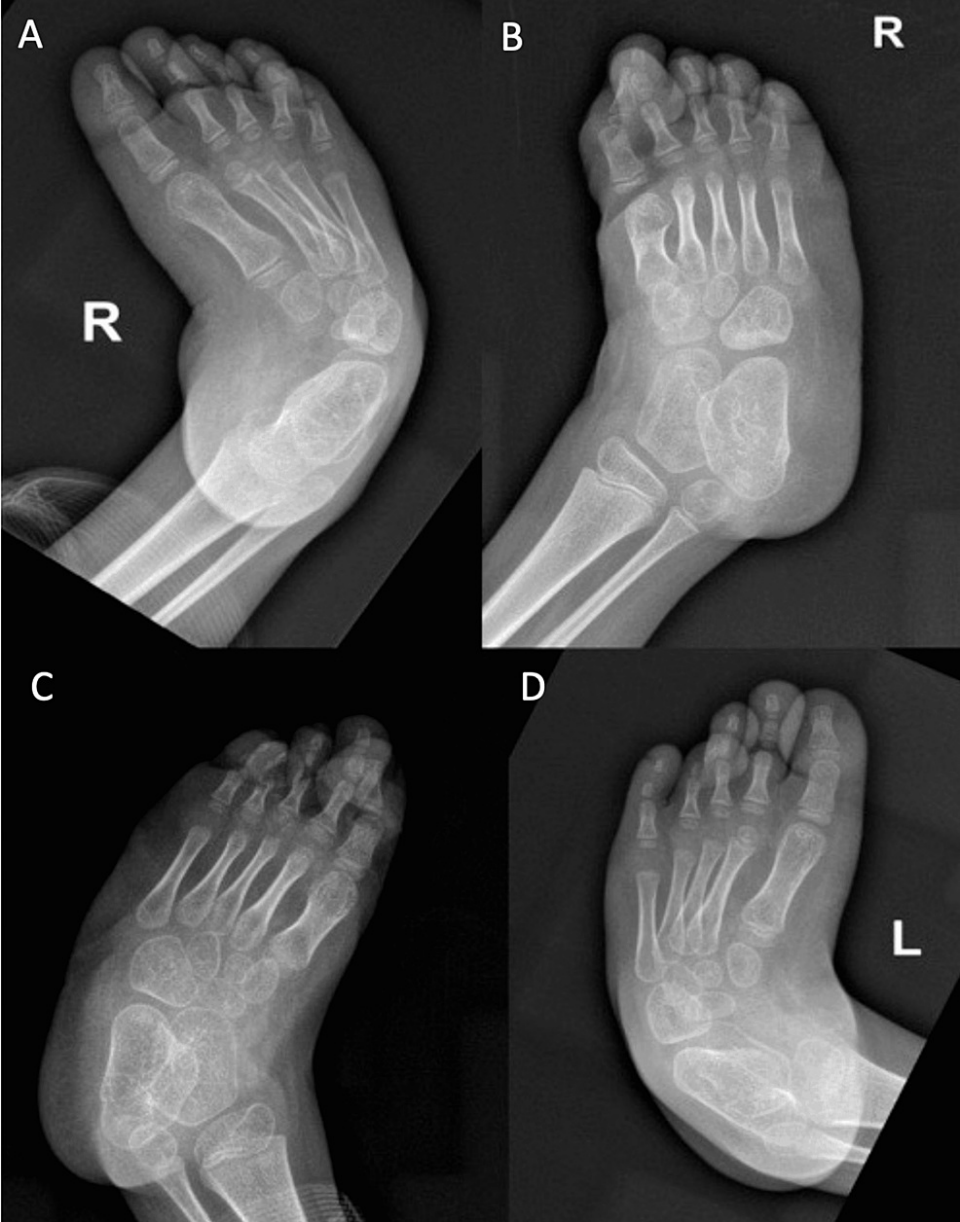
Preoperative x-ray images of Case 2 (A) Anteroposterior (AP) view of the right foot preoperatively. (B) Oblique view of the right foot preoperatively. (C) Oblique view of the left foot preoperatively. (D) AP view of the left foot preoperatively.

We present a five-year-old Saudi boy, known to have central sleep apnea, developmental delay, MMC post-repair, type 2 Chiari malformation post-shunt placement in 2017 with subsequent changes of the shunts due to infections, post-suboccipital craniectomy, C1-2 laminectomy, bilateral hip dislocations, and bilateral club feet. He spent a long time in the ICU since birth. When he was brought to our clinic, he had no formal physical therapy done nor was enrolled in school. He was able to crawl, was ambulant above both knees, and had functioning knee extensors and bilateral insensate and rigid feet with tight tendoachilles and cavus but correctable forefoot adduction. His knees showed no deformity, his left hip abduction was limited, but his right hip showed reduction in hip abduction. Initially, bilateral Achilles tenotomy and serial casting were attempted to achieve plantigrade, shoeable feet. During his follow-up visits, he was then switched to Denis-Brown shoes, which consist of a straight aluminum bar that has two straight-border, open-toe, high-top shoes attached to it. The distance between both heels is equal to the width of the child's shoulders. He was then scheduled for surgery for his bilateral hip dislocations, was prescribed bilateral knee-ankle-foot-orthosis, and referred for rehabilitation. In April 2019, he underwent bilateral hip adductor tenotomy, bilateral open reduction, bilateral acetabuloplasty, bilateral femoral shortening, and right flexor release. He tolerated the procedure well and was admitted for overnight observation to the pediatric intensive care unit. After his safe discharge and during his follow-up, he was scheduled for bilateral foot correction, which he underwent in November 2020, with the addition of bilateral femoral plates removal (see Figure 4).

**Figure 5:**
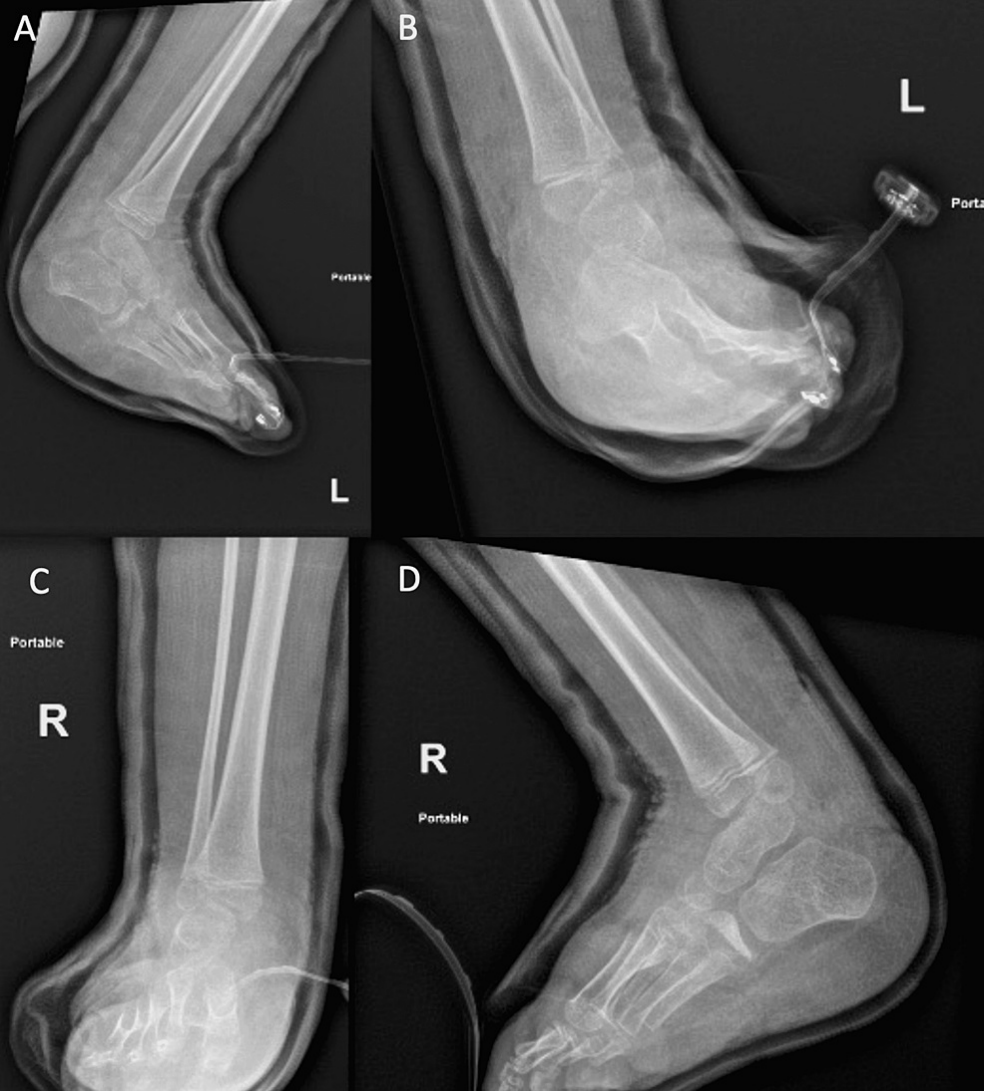
Immediate postoperative x-ray images of Case 2 (A) Lateral x-ray image of the left foot. (B) Anteroposterior (AP) x-ray image of the left foot. (C) AP x-ray image of the right foot. (D) Lateral x-ray image of the right foot.

Unfortunately, we found no record of his preoperative clinical images. Intraoperatively, percutaneous release was done along with a small medial incision for the cuboid osteotomy, similar to the case mentioned above. Both femoral plates were removed, and bilateral foot casting was applied (see Figure 5).

**Figure 6:**
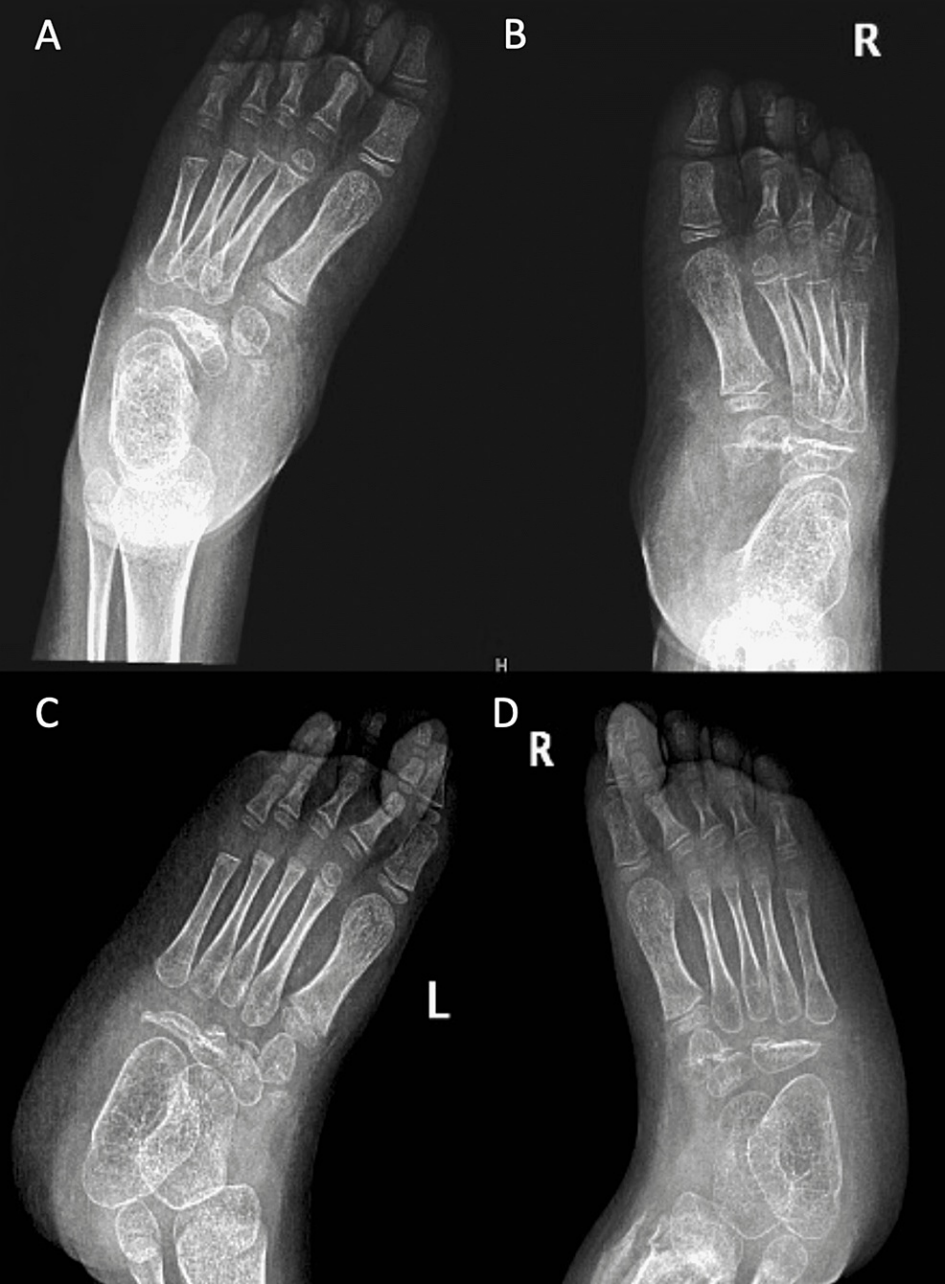
Three months postoperative x-ray images of Case 2 (A) Anteroposterior (AP) view of the left foot. (B) AP view of the right foot. (C) Oblique view of the left foot. (D) Oblique view of the right foot.

**Figure 7:**
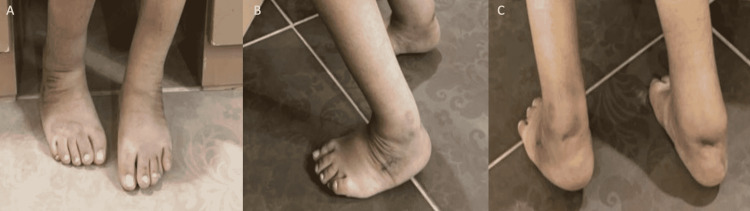
Postoperative clinical appearance of the bilateral foot deformity in Case 2 after three months (A) Anterior view postoperatively of the foot deformity after corrective surgery. (B) Lateral view postoperatively of the foot deformity after corrective surgery. (C) Posterior view postoperatively of the foot deformity after corrective surgery.

He was admitted once more to the pediatric intensive care unit for observation and was discharged home safely after he was safely transferred to the ward. During his follow-up visits, he was doing well. The bilateral foot casts were removed, and AFO was applied bilaterally at three months postoperatively (see Figures 6, 7).

During his most recent visit, approximately at six months postoperatively, the child is able to crawl, stand, and take a few steps with assistance. He is still following as an outpatient in our clinic, but due to the COVID-19 circumstances, we were following him virtually over the phone. As per both parents, he was doing well. He is scheduled for a physical visit to our clinic soon.

## Discussion

The presence of a rigid teratologic clubfoot in MMC is thought to reach up to 40% [7]. In those patients, previous studies using conservative measures have yielded poor results. These severe deformities are commonly seen since birth and are often more resistant to well-conducted non-surgical treatment [16]. It is important to highlight that clubfeet will never be strictly normal, regardless of treatment [16].

Generally, the development of non-surgical options for management has decreased the need for surgical intervention in clubfoot. Nonetheless, severe deformities, such as those seen in MMC, are more resistant to conservative measures and are likely to require surgery [16]. Several studies highlighted the low efficiency of repeat casting when the first trial of Ponseti casting failed as it increases the risk of relapse, with failure rates reaching 90% [18,20]. These studies also unanimously agreed that surgery should not be avoided when necessary [16]. Despite this, serial casting in patients who were undergoing surgical correction was still used to stretch the skin and soft tissue as much as possible prior to proceeding to the surgical intervention [7,15,21].

The use of the targeted minimally invasive percutaneous posteromedial release of residual clubfoot in patients with rigid clubfoot such as those with MMC has not been previously described in the literature. However, there have been several papers in the literature that addressed several methods and modifications of the posteromedial release [7,12,16,22-26].

Flynn et al. [7] described an approach of a radical posteromedial release without internal fixation of the talonavicular or subtalar joints. They obtained good results in 62.5% of patients (45 out of 72), and only 12.5% of patients (nine out of 72) had poor results. In their study, no statistical difference was noted between older children and those who underwent this procedure between the ages of 12 and 18 months. Functional motor level of involvement did not correlate with prognosis [7].

Walker [25] involved 35 feet in their study. They treated the hindfoot deformity by a combination of stretching, splinting, and a minor posterior release when deemed necessary. Out of the 35 feet that were manipulated, 21 feet obtained good results after posterior release, two were said to have been good after subcutaneous Achilles tenotomy, and seven were corrected with manipulation and strapping alone. In that study, the extensive medial releases described included a rotation flap with extensive tenotomies [25].

Sharrard and Grosfield [12] described 78 equinovarus feet that required 122 soft tissue operations, 30 tendon transfers as well as 38 bony procedures. There was a failure rate of 22% requiring operative revisions, seven skin breakdowns, and five superficial infections [12].

de Carvalho Neto et al. [26] reported 63% of good results for 63 feet undergoing an extensive posteromedial lateral release. In 21 patients, a K-wire technique was used to derotate the talus in the ankle mortise, which led to 76% of good results. Moreover, the low lumbar level had 27 good feet, five fair, and two poor. In the poor group, 10 feet required additional surgery. Four feet did not undergo salvage procedures due to having a high neurosegmental level and mental retardation [26].

Bocahut et al. [16] developed a surgical approach that reproduced the chronological steps of performing forefoot derotation before correction of the hindfoot equinus. This approach was not similar to Turco’s or Carroll’s procedures in terms of extensive soft tissue release, despite being considered a medial to posterior approach. Instead, this approach utilized a medial approach and followed soft tissue release according to the deformity. It always consisted of an anteromedial release followed by a posterolateral release when needed to correct the equinus. The deformity was said to be reduced when the navicular bone was in front of the talar head. If residual equinus remained after Achilles Z-lengthening, a posterior ankle arthrotomy was performed through the same approach. After that, the lateroposterior node was dissected, but no posterior subtalar arthrotomy was done. The talocalcaneal ligament was preserved to avoid overcorrection with lateral translation and valgus as a result of destabilization of the subtalar joint. Then, the Achilles tendon and the tibialis posterior were sutured with the foot in a neutral position. To maintain the position, a K-wire under fluoroscopic guidance was utilized. It would run from the first metatarsal bone to the talus and across the talonavicular joint. Then patients were immobilized and kept non-bearing for six weeks in a long-leg cast flexed at 90 degrees to maintain the ankle and foot dorsiflexion. One hundred and thirty-seven patients with severe clubfeet (Dimeglio score of 12.0) were followed until skeletal maturity was achieved. The surgical approach described in their study resulted in a mean International Clubfoot Study Group (ICFSG) score of 4.3 at a mean follow-up of 10.8 years, with only 12% requiring revision surgery [16].

Other surgical options described in the literature include a talectomy, which is preferred for the older patient population and is implemented as a salvage procedure for a severe, non-braceable, rigid equinovarus foot with a previously failed radical posteromedial release [7].

Complications highlighted in the literature with the radical posteromedial release included incomplete correction of the deformity, deformity recurrence, and wound-healing problems ranging from minor skin ulcers to wound dehiscence requiring skin grafting [7].

There are several limitations to our current study and our proposed procedure. First, it is a report of two cases only. Second, the possibility of incomplete release of the addressed structures and recurrence are to be noted. Third, the lack of participants who underwent this form of posteromedial release is an issue. Furthermore, we still do not have an adequate period of follow-up to generalize such outcomes. As far as our knowledge, there are not any long-term studies involving adult patients and the function of the feet after surgery. In addition, the involved patients have also undergone other procedures, which may affect the overall prognosis of the condition of their feet. Moreover, the level of the MMC may affect the results of such procedures; therefore, more patients are needed to generalize our findings.

## Conclusions

The relatively minimally invasive technique that was described in our study has shown excellent results in both patients in achieving plantigrade, shoeable feet. It is a promising method of management for those with clubfeet due to MMC. We do, however, suggest recruiting more patients as well as obtaining a longer period of follow-up. This minimally invasive method of release may also be trialed on patients with idiopathic clubfoot as well.
